# Screening and Identification of Host Factors Interacting with the Virulence Factor P0 Encoded by Sugarcane Yellow Leaf Virus by Yeast Two-Hybrid Assay

**DOI:** 10.3390/genes14071397

**Published:** 2023-07-03

**Authors:** Kai-Li Liang, Jing-Ying Liu, Ying-Ying Bao, Zhi-Yuan Wang, Xiong-Biao Xu

**Affiliations:** 1 Guangxi Key Laboratory for Sugarcane Biology, Guangxi University, Nanning 530004, China; liangkelly07@163.com (K.-L.L.); 15678302282@163.com (J.-Y.L.); baoyingying2001@163.com (Z.-Y.W.); 2 College of Agriculture, Guangxi University, Nanning 530004, China; w1274105733@163.com; 3 State Key Laboratory for Conservation and Utilization of Subtropical Agro-Bioresources, Guangxi University, Nanning 530004, China

**Keywords:** sugarcane yellow leaf virus, P0 protein, pathogenesis, yeast two-hybrid, interaction

## Abstract

Sugarcane yellow leaf virus (SCYLV), a member of the genus *Polerovirus* in the family *Luteoviridae*, causes severe damage and represents a great threat to sugarcane cultivation and sugar industry development. In this study, inoculation of *Nicotiana benthamiana* plants with a potato virus X (PVX)-based vector carrying the SCYLV P0 gene induced typical mosaic, leaf rolling symptoms and was associated with a hypersensitive-like response (HLR) necrosis symptom, which is accompanied with a systemic burst of H_2_O_2_ and also leads to higher PVX viral genome accumulation levels. Our results demonstrate that SCYLV P0 is a pathogenicity determinant and plays important roles in disease development. To further explore its function in pathogenic processes, a yeast two-hybrid assay was performed to screen the putative P0-interacting host factors. The recombinant plasmid pGBKT7-P0 was constructed as a bait and transformed into the yeast strain Y2HGold. The ROC22 cultivar (an important parental resource of the main cultivar in China) cDNA prey library was constructed and screened by co-transformation with the P0 bait. We identified 28 potential interacting partners including those involved in the optical signal path, plant growth and development, transcriptional regulation, host defense response, and viral replication. To our knowledge, this is the first time we have reported the host proteins interacting with the P0 virulence factor encoded by sugarcane yellow leaf virus. This study not only provides valuable insights into elucidating the molecular mechanism of the pathogenicity of SCYLV, but also sheds light on revealing the probable new pathogenesis of *Polerovirus* in the future.

## 1. Introduction

Sugarcane (*Saccharum officinarum* L.) is a key sugar-producing and bioenergy crop worldwide in the tropical and subtropical regions. However, sugarcane production is adversely affected by several pathogens and other factors, among which sugarcane yellow leaf virus (SCYLV)—the causal agent of sugarcane yellow leaf disease, which belongs to the genus *Polerovirus* in the family *Luteoviridae*—poses a great threat to sugarcane cultivation and sugar production [1,2].

The SCYLV genome encodes six open reading frames (ORF0~ORF5), and the ORF0 encodes a 30.2 kDa P0 protein, which inhibits RNA silencing and participates in viral expression [3,4]. A large number of studies have shown that the P0 protein of the *Polerovirus* is a silencing suppressor [5,6,7], but its mechanism still remains ambiguous. However, the P0 proteins encoded by different species of *Polerovirus* show low sequence identity and the activity of RNA silencing suppressor (RSS) also exhibits a great difference. Moreover, the RSS activity may also have a large difference among different isolates of the same viral species. The P0 obtained in this study was verified to be a strong RSS (Figure S1), which is consistent with previous reports [3]. P0 deletion mutants of Beet mild yellowing virus (BWYV) and Potato leaf roll virus (PLRV) caused a significantly reduced or even undetectable viral accumulation when infiltrated by agroinoculation, and the P0 protein is thus considered necessary for successful viral infection [8,9].

To counter host defense, plant viruses encode several virulence factors to neutralize host immunity by interacting with specific host factors. The orthologs of SKP1 (S-phase kinase-related protein-1) from *Arabidopsis thaliana* were proved to interact with Cucurbit aphid-borne yellows virus (CABYV) P0 by yeast two-hybrid (Y2H) screening, and the P0–SKP1 interaction was indispensable for efficient BWYV infection [10]. And further experiments have shown that the F-box-like motif of most *Polerovirus* P0 proteins can target and degrade host ARGONAUTE1 (AGO1) to suppress RNA silencing [11,12]. It is worth noting that the P0 protein of PLRV failed to interact with SKP1 when tested by Y2H systems, which indicates that the degradation of AGO1 may be carried out by an alternative mechanism [13]. However, there was no evidence of direct interaction between P0 and AGO1 in subsequent experiments [11,14]. Previous experiments have proven that the P0-mediated degradation of AGO1 is insensitive to proteasome inhibitors, which is inconsistent with the view that AGO1-targeted P0 undergoes ubiquitination and proteasome-dependent degradation, suggesting that other cellular proteases may be involved in this process [11]. Later studies have shown that PLRV P0 can mediate the degradation of various AGOs and interact with the PAZ (Piwi, Argonaute, and Zwille/Pinhead) domain and adjacent upstream region of the AGO-1 protein transiently or indirectly in vitro [12,14], which inhibits the formation of miRNA-RISC and ultimately leads to AGO1 degradation, thus inhibiting RNA silencing [14]. In addition, Derrien and colleagues showed that the autophagy pathway was involved in P0-mediated AGO1 degradation [15]. In conclusion, the determinate mechanism of the P0 protein exerting its silencing suppressor function remains to be confirmed.

The transient expression evidence supported the fact that the P0 protein encoded by several members of *Polerovirus* could cause leaf necrosis in *N. benthamiana* and *N. glutinosa* [3,14,16]. The phenomenon of leaf necrosis has a dose–effect response and may also be related to the systemic silencing suppressor ability [3,14]. It is suggested that the leaf necrosis symptom may be a resistance reaction induced by the host recognition of the P0 protein. No DNA ladder phenomenon was found in SCYLV P0-induced necrosis leaves. So, it is considered that P0-induced necrosis is not a programmed cell death (PCD) or hypersensitive reaction (HR) [3]. However, there are precedents to show that the DNA ladder does not appear in the process of PCD of plant cells [17], which means the existence of the DNA ladder cannot be regarded as reliable evidence to identify the type of necrosis caused by P0. The roles of P0 still remain controversial.

The pathogenicity of plant viruses is influenced by the symptom determinants encoded by viruses, which play significant roles during the infection. SCYLV P0 has been proven to have RSS activity and induce necrosis, which indicates it to be a potential pathogenic factor, but little is known about its detailed pathogenic mechanism and the host factors involved. To further explore the functions of P0 in viral pathogenic processes, a yeast two-hybrid system was performed in this study to screen host factors interacting with SCYLV P0 from the cDNA of library of *S. officinarum* ROC22. Notably, the function of the host proteins is discussed. This study offers an experimental basis for studying the molecular mechanism of pathogenicity of the P0 protein and virus–host interaction and lays a foundation for further illustrating the pathogenesis of SCYLV and *Polerovirus*.

## 2. Materials and Methods

### 2.1. Sequence Analysis

The first-strand cDNA of SCYLV was obtained by reverse transcription polymerase chain reaction (RT-PCR) using HiScript III 1st Strand cDNA Synthesis Kit (+gDNA wiper) (Cat. No.: R312-01, Vazyme Biotech Co., Ltd., Nanjing, China). The complete genome sequence of SCYLV was amplified by using SCYLV degenerate primers (seen in Supplementary Table S1) and Phanta EVO HS Super-Fidelity DNA Polymerase (Cat. No.: P504-d1, Vazyme Biotech Co., Ltd., Nanjing, China), according to manufacturer’s instructions. The product was separated on a 1.0% agarose gel and verified by Sanger sequencing, and the ORF0 was predicted and identified using Open Reading Frame Finder software at the NCBI. Multiple sequence alignment of SCYLV P0 and other *Polerovirus* P0 amino acid sequences was performed through MegAlign program by using DNASTAR software. Phylogenetic analysis based on the amino acid sequences of proteins P0 from 18 *Polerovirus* was performed by using MEGA 7.0 software via the Neighbor-Joining Method with 1000 bootstrap replications. The names and GenBank accession numbers of the viruses are listed in Supplementary Table S2.

### 2.2. Plant Materials and Growth Conditions

*N. benthamiana* seedlings were grown in an insect-free growth chamber at 25 ± 2 °C and 60% relative humidity under a 16 h light/8 h dark photoperiod. Leaves and stalks of healthy ROC22 sugarcane cultivar were collected from Nanning, Guangxi Zhuang Autonomous Region. The samples were collected and mixed equably and frozen rapidly in liquid nitrogen, and stored at −80 °C. The total sample was collected for cDNA library construction.

### 2.3. Plasmid Construction

The full length of the P0 gene was amplified from the SCYLV (not submitted yet) using the primer pair SCYLV/P0/BamH I/F and SCYLV/P0/Sal I/R (Supplementary Table S1) and Phanta EVO HS Super-Fidelity DNA Polymerase (Cat. No.: P504-d1, Vazyme Biotech Co., Ltd., Nanjing, China). The PCR profile was adjusted as an initial denaturation at 95 °C for 3 min, denaturation at 95 °C for 30 s, annealing at 56 °C for 30 s, extension at 72 °C for 23 s, and then final extension at 72 °C for 5 min followed by 30 cycles. To construct the PVX-P0 heterologous expression recombinant plasmid, the full-length sequence of P0 (771 bp) was further PCR-amplified using primer pair SCYLV /P0/Cla I/F and SCYLV /P0/Sal I/R (Table S1). Then, the PCR products were digested with the restriction endonucleases Cla I (Cat. No.: FD0143, Thermo Fisher Scientific Inc., Waltham, MA, USA) and Sal I (Cat. No.: FD0644, Thermo Fisher Scientific Inc., Waltham, MA, USA), and subcloned into the same enzyme-digested PVX-based vector to generate PVX-P0.

For the Y2H screening assays, the pGBKT7-P0 vector was constructed and the full-length sequences of P0 were PCR-amplified using primer pair SCYLV/P0/BamH I/F and SCYLV/P0/Sal I/R (Table S1). Then, the PCR products were digested with the restriction enzymes BamH I (Cat. No.: FD0054, Thermo Fisher Scientific Inc., Waltham, MA, USA) and Sal I, and then inserted into the pGBKT7 vector (Cat. No.: 630443, Takara Biomedical Technology Co., Ltd., Beijing, China), which was treated with the same enzymes to generate the pGBKT7-P0 bait plasmid. The recombinant plasmid was verified by PCR and Sanger Sequencing.

### 2.4. Agroinfiltration Assays

For inoculation of plants, the recombinant PVX-P0 plasmid was transformed into *Agrobacterium tumefaciens* strain GV3101 by electroporation [18]. The transformants were cultured on Yeast Extract Peptone Medium (YEP) solid medium supplemented with rifampicin (50 μg · mL^−1^) and kanamycin (50 μg · mL^−1^) at 28 °C for 2 days. Then, the *A. tumefaciens* was transferred into YEP liquid medium containing the corresponding antibiotics and cultivated overnight at 230 rpm in a 28 °C shaker. The cultures were centrifuged and resuspended in infiltration buffer (10 mM MES, pH 5.6; 200 μM acetosyringone; 10 mM MgCl_2_) to OD_600_ = 0.6, then infiltrated into *N. benthamiana* plants at the 4–6-leaf stage using a sterile needle-free syringe as described previously [19].

### 2.5. H_2_O_2_ Detection in Plants

The accumulation of H_2_O_2_ was detected in systemic leaves by using the 3,3′-Diaminobenzidine (Cat. No.: D12384, Sigma-Aldrich, Merck KGaA, Darmstadt, Germany) staining method as described previously [20] and had some modifications. The systemic leaves of *N. benthamiana* plants were collected and photographed, then soaked in the DAB staining solution (1 mg · mL^−1^, pH 3.8) and kept in the dark at 25 °C for 10 h, followed by blanching in boiled 96% ethanol for 5–10 min until the chlorophyll was completely bleached out. The corresponding leaves were then kept in 70% ethanol and photographed by using a Canon EOS 90D digital camera (Canon Co., Ltd., Beijing, China).

### 2.6. Protein Extraction and Western Blot analysis 

Total protein was extracted from inoculated leaves using protein extraction buffer (50 mM Tris-HCl, pH 6.8; 4.5% SDS; 7.5% 2-mercaptoethanol; 9 M Urea). The total proteins were separated by 10% sodium dodecyl sulphate–polyacrylamide gel electrophoresis (SDS-PAGE) and transferred onto a nitrocellulose (NC) membrane. The anti-PVX CP monoclonal antibody was used at a 1:5000 dilution and incubated with the membranes at 37 °C for 2 h. Subsequently, the membranes were soaked in 5% skim milk solution containing horseradish peroxidase (HRP)-conjugated goat anti-mouse IgG secondary antibody. Finally, after rinsing in 1 x PBST several times, the membranes were used for the detection of chemiluminescence by using a Chemiluminescence imaging system (ImageQuant LAS 500, Cytiva, Marlborough, MA, USA).

### 2.7. cDNA Expression Library Construction and Quality Assay

The total RNA from the whole tissue of sugarcane (*S. officinarum*) ROC22 was extracted using RNAiso Plus reagent (Cat. No.: 9108, Takara Biomedical Technology (Beijing) Co., Ltd, Beijing, China) and dissolved in Nuclease-free ddH_2_O; the concentration, purity, and integrity of the total RNA were analyzed using both the NanoPhotometer N50 instrument (IMPLEN, Munich, Germany) and 1.5% agarose gel electrophoresis (AGE). More than 300 µg of total RNA with ratios of A260/A280 between 1.8 and 2.2 and a concentration above 150 ng · µL^−1^ was used for subsequent analysis.

The mRNA was purified by Oligo (dT) magnetic beads and was reverse-transcribed to single-stranded cDNA. The above single-stranded cDNA was used as a template to amplify double-stranded cDNA in three segments and purified by DNA Clean Beads. The purified double-stranded cDNA was averaged, and then small segments were removed by Clontech CHROMA SPINTM+TE-1000 Columns (Cat. No.: 636079, Takara Biomedical Technology (Beijing) Co., Ltd, Beijing, China).

The double-stranded cDNA was homologously recombined with the pGADT7 linearized vector, and then the product was shocked into the *Escherichia coli* TOP10 competent cells and then transferred to Super Optimal broth (SOC) liquid medium and cultured at 37 °C for 1 h. Dilute (1000-fold) 10 µL bacterial solution and 100 µL of bacterial solution were cultured on Luria–Bertani (LB) solid medium (containing 100 µg · mL^−1^ ampicillin) overnight, and glycerol was added to the remaining bacterial solution (the final concentration was 20%) as library bacterial solution.

Twenty-four individual clones were chosen for PCR detection by using universal primers for the pGADT7 vector (T7-F:5′-TAATACGACTCACTATAGGGCGAG-3′; 3′AD:5′-GCACGATGCACAGTTGAAG-3′). The library plasmids were extracted from library bacterial solution by using the High-Concentration Plasmid Extraction Kit (Cat. No.: DP116, TIANGEN Biotech (Beijing) Co., Ltd., Beijing, China) and the obtained cDNA yeast library was stored at −20 °C.

### 2.8. Assays of Auto-Activation and Toxicity of the Bait Plasmid

It is important to test the bait protein for autoactivation prior to Y2H screening. The recombinant pGBKT7-P0 plasmid and pGBKT7 empty plasmid were transformed into Y2HGold yeast-competent cells, respectively. Transformants were incubated on SD/-Trp plates for 3–5 days at 30 °C to test the toxicity of P0 protein. If colonies expressing P0 were similar to those expressing the pGBKT7 empty vector, the bait was verified to be non-toxic. To test the auto-activation activity of P0, the pGBKT7-P0 plasmid and pGBKT7 empty vector were co-transfected with pGADT7 plasmid into Y2HGold yeast competent cells, respectively. Transformants were incubated on Double Dropout SD/-Trp/-Leu (DDO) agar plates for 3–5 days at 30 °C. Colonies were then picked out and transferred to higher-stringency agar plates, as Triple dropout SD/-Leu/-Trp/-His (TDO) or Quadruple Dropout SD/-Ade/-His/-Leu/-Trp/X-α-Gal (QDO/X). pGADT7-T + pGBKT7-Lam (negative control) were co-transformed and grown on TDO and QDO/X plates and pGADT7-T + pGBKT7-53 as a positive control. The color and size of the colonies were observed. Bait plasmids with no self-activation or toxicity were used for the subsequent yeast two-hybrid screening.

### 2.9. Yeast Two-Hybrid Screening with Co-Transformation

To screen out sugarcane host proteins that interact with SCYLV P0, the ROC22 cDNA prey plasmids library and pGBKT7-P0 were co-transformed into Y2HGold by the PEG/LiAC method. The co-transformants were cultured on SD/-Leu/-Trp/-His/X-α-Gal (TDO/X) agar plates at 30 °C for 5–7 days. Colonies with growth and blue were picked out and streak-cultured to a higher-stringency QDO/X agar plate. To determine the fragment size of each potential interacting prey plasmid, the universal primers of the prey plasmid pGADT7 were used for PCR amplification identification (Table S1).

### 2.10. Validation of the Screened Interactions

To validate the screened interactions, each of the potential positive prey plasmids was again co-transformed with pGBKT7-P0 into Y2HGold. The detailed steps were as follows. Each screened putative positive prey plasmid was extracted from the corresponding yeast clone by using a Yeast Plasmid Extraction Kit (Solarbio, D1160, Beijing Solarbio Science & Technology Co., Ltd., Beijing, China). To separate the probable multi-prey transformants and raise the concentration of plasmids, the obtained plasmids were transformed into *E. coli* DH5α and cultured on LB (containing 100 µg/mL of ampicillin) agar plates. The positive clones were verified by PCR and followed by plasmid extraction by using the FastPure Plasmid Mini Kit (Vazyme, DC201-01). Subsequently, each inferred positive prey and pGBKT7-P0 bait plasmid were co-transformed into Y2HGold and planted on QDO/X agar plates to demonstrate the interactions. Under these steps of confirmation, the blue colonies indicate true positive interactions.

### 2.11. Function Analysis of True Positive Prey

The validated true positive prey plasmids were sent to Sangon Biotech (Shanghai, China) Co., Ltd. for sequencing. The online NCBI and Sugarcane Genome Hub database (https://sugarcane-genome.cirad.fr) BLAST sequences were used to analyze the function of positive preys.

## 3. Results

### 3.1. Phylogenetic Analyses and Domain Architecture of Sugarcane-Yellow-Leaf-Virus-Encoded P0

**Figure 1:**
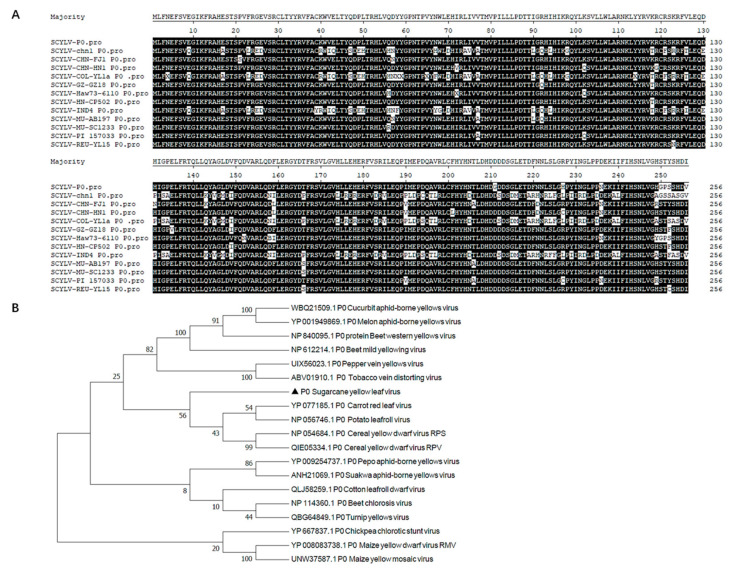
Multiple sequence alignment and phylogenetic analysis of P0 proteins. (**A**) Multiple sequence alignment of P0 proteins from different sugarcane yellow leaf virus isolates. (**B**) Phylogenetic tree of P0 proteins. The amino acid sequences of P0 from 18 poleroviruses were aligned using the Neighbor-Joining method with the MEGA7.0 program with bootstrap 1000 replicates, and the GenBank accession numbers are shown in the phylogenetic tree.

The full length of SCYLV ORF0 is 771 nucleotides (nt) and encodes a small protein named P0 of 256 amino acids (aa). Among the 14 sequenced representative isolates of SCYLV, the length of P0 is between 256 and 257 aa. The multiple alignment shows that all the P0 amino acid sequences exhibit high sequence similarity (Figure 1A), which indicates that the SCYLV P0 protein is quite conserved among different isolates. To examine the evolutionary relationships of P0 sequences from different *Polerovirus* species, a phylogenetic dendrogram of the complete P0 amino acid sequences of 18 representative *Polerovirus* species was generated by using the Neighbor-Joining method with the MEGA7.0 software. As shown in Figure 1B, the P0 proteins of poleroviruses showed low sequence homology, and the P0 of SCYLV clustered in a single branch, which shows a closer genetic relationship to those of Carrot red leaf virus (CRLV) and Potato leafroll virus (PLRV), and the P0 of these three viruses clustered in the same clade.

### 3.2. P0 Is a Virulence Determinant and Elicits a Hypersensitive-Like Response (HLR) in N. benthamiana

**Figure 2:**
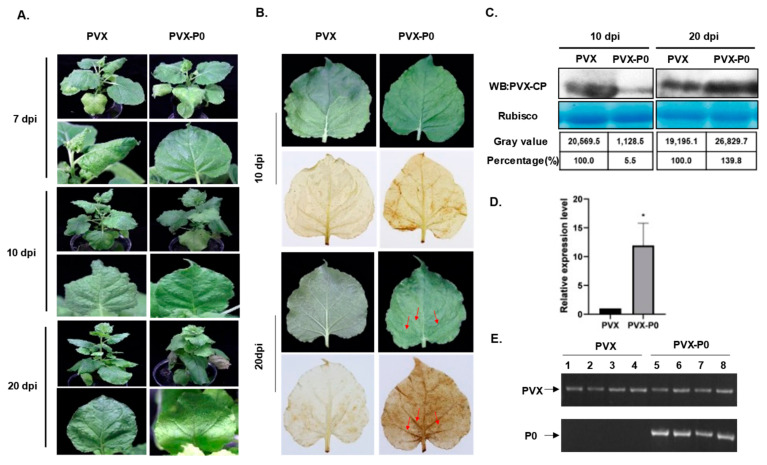
Symptoms exhibited by plants following inoculation with Potato virus X (PVX) or PVX-P0. (**A**) Symptoms elicited on *N. benthamiana* plants at 7 days post inoculation (dpi), 10 dpi, and 20 dpi infected with PVX or PVX-P0. (**B**) Mosaic symptoms and necrotic lesions induced by PVX-P0. *N. benthamiana* plants were infected with PVX or PVX-P0. Upper infected leaves were photographed directly at 10 dpi and 20 dpi (upper panels) or photographed after 3,3′-diaminobenzidine (DAB) staining (lower panels). The red arrowheads indicate the necrotic lesions. (**C**) Western blot (WB) analysis of PVX coat protein (CP) accumulation in plants infected with PVX or PVX-P0. Total protein was extracted from newly emerged leaves. Coomassie-brilliant-blue-stained Rubisco large subunit was used as a loading control, and the anti-PVX CP monoclonal antibody was used. The gray values of the blot bands were analyzed by using the ImageJ software, and the relative content of CP accumulated in PVX-infected plants was set as 100%. (**D**) RT-qPCR detection of relative expression levels of PVX CP in PVX- and PVX-P0-infected plants at 20 dpi. T-tests were performed to identify statistically significant differences (* *p* < 0.05). Three individual *N. benthamiana* leaf samples were used for RNA extraction and subsequent RT-qPCR detection. All experiments were repeated at least three times. (**E**) Detection of PVX and P0 by RT-PCR using PVX and SCYLV P0-specific primers, respectively.

Since the P0 protein of SCYLV has been proven to be a viral encoded RNA silencing repressor (VSR) [3], in order to determine whether P0 has potential functions in viral infection, P0 was ectopically expressed via the Potato virus X (PVX)-based vector in *N. benthamiana* plants. *A. tumefaciens* harboring PVX-P0 and PVX empty vectors was inoculated into the 5-6-leaf stage *N. benthamiana* seedlings by agroinfiltration, respectively. During the first 7 days post inoculation (dpi), the plants infected with PVX showed an obvious mosaic phenotype (Figure 2A, upper panels). Starting at about 10–12 dpi, plants inoculated with PVX began to recover, with a gradual disappearance of mosaic and veinal chlorosis phenotypes by 20 dpi (Figure 2A, intermediate panel). However, the constant expression of P0 resulted in leaf-rolling and chlorosis symptoms after 10 dpi. These symptoms could be observed in the upper systemic leaves of all 12 *N. benthamiana* plants inoculated with PVX-P0 at 20 dpi (Figure 2A, lower panel). In addition, distinct necrotic phenotypes were observed in plants infected with PVX-P0 at late stages of infection, but the PVX-inoculated plants failed to induce such a necrotic symptom (Figure 2B). To confirm whether the necrosis is due to the accumulation of H_2_O_2_, we analyzed the content of H_2_O_2_ in systemic leaf tissues of PVX- and PVX-P0-infected plants by using the 3,3′-diaminobenzidine (DAB) staining method. In the case of H_2_O_2_, DAB polymerized to insoluble sediments and generated a visible deep brown color after ethanol bleaching. High concentrations of H_2_O_2_ were found in systemic leaves of PVX-P0-infected plants, while no H_2_O_2_ was detected in PVX-inoculated control tissues (Figure 2B). To explore the relationship between P0-induced severe symptoms and PVX accumulation, Western blots were conducted to detect the CP expression levels of both PVX and PVX-P0-inoculated plants at 10 and 20 dpi (Figure 2C). The content of the PVX CP protein accumulated to a higher concentration when compared with that of PVX-P0-infected plants at 20 dpi, but the result was the opposite at 10 dpi. The relative transcription levels of PVX CP were also analyzed at 20 dpi by RT-qPCR using the PVX-CP-specific primers, and we found that the relative expression level of CP in PVX-P0-inoculated plants was much higher than that of PVX-infected plants (Figure 2D). These results suggest that P0 is a virulence factor that assists in the replication of PVX in the late stage of infection (Figure 2C,D). Total RNA of the inoculated plants was extracted, the presence of PVX and P0 was detected by RT-PCR using specific primer pairs, and the PVX vector could be detected in all inoculated plants, while P0 could only be detected in the plants inoculated with PVX-P0 (Figure 2E), which indicates P0 to be the symptom inducer. The above results indicate that SCYLV P0 is a symptom determinant that can trigger an HLR in *N. benthamiana* when expressed from PVX-P0.

### 3.3. Construction of cDNA Library and pGBKT7-P0 Bait Plasmid

The mRNA from ROC22 sugarcane aboveground parts showed excellent quality (Figure S2A). The capacity of the yeast two-hybrid cDNA library is about 1.36 × 10^8^ clones (Figure S2B) and the average length of the inserted fragments of 24 representative clones was about 1 kb (Figure S2C). The results suggested that the quality of the sugarcane cDNA library was acceptable and can be adopted for the subsequent yeast two-hybrid screening. The concentration of the prey plasmids was 670 ng · µL^−1^ and the total quantity was 335 µg. The ORF0, which is 771 bp in length, was amplified by using the SCYLV-infected sugarcane cDNA as the template. Restriction endonuclease digestion and sequencing were performed to confirm the successful construction of the pGBKT7-P0 bait plasmid. 

### 3.4. Assay Results of Auto-Activation and Toxicity of the Bait Plasmid

**Figure 3:**
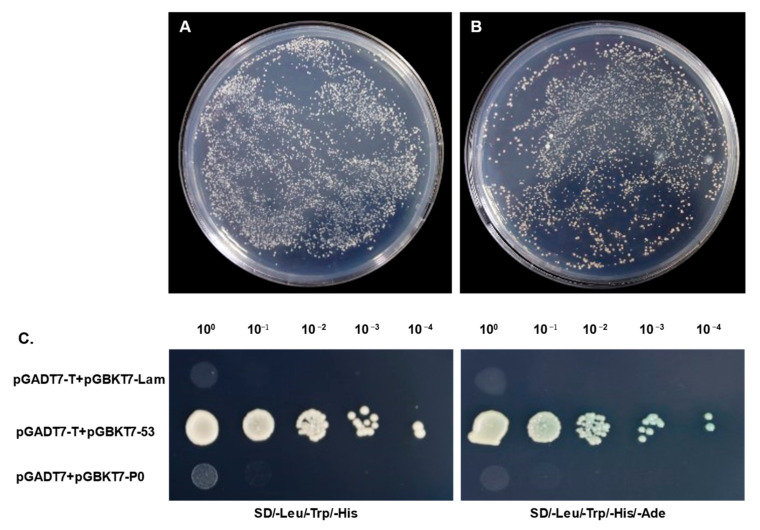
Autoactivation and toxicity of pGBKT7-P0 in yeast. (**A**) The toxicity of pGBKT7 vector and pGBKT7-P0 prey plasmid (**B**). (**C**) The transformed Y2HGold yeast cells grew on SD/-Leu/-Trp/-His (TDO) or SD/-Leu/-Trp/-His/-Ade/X-α-Gal (QDO/X) agar plates. The plasmid pGBKT7-53 or pGBKT7-Lam was co-transformed with pGADT7-T into Y2HGold yeast cells, which was used as the positive or negative control, respectively.

The co-transformation of the pGBKT7-P0 bait plasmid and pGADT7 empty vector into the Y2HGold strain was conducted and the transformants were cultured on SD/-Leu/-Trp (DDO), SD/-Leu/-Trp/-His (TDO), or SD/-Ade/-His/-Leu/-Trp/X-α-Gal (QDO/X) agar plates to detect any auto-activation activity. The colonies containing pGBKT7-P0 grew on neither TDO nor QDO/X plates, while the colonies of the positive control turned blue, indicating that pGBKT7-P0 had no activity of auto-activation. There were no significant differences between the size of the P0-transforned Y2H colonies and that of the pGBKT7 control, which proved that the bait was non-toxic. As a result, the pGBKT7-P0 bait plasmid was capable of adoption in the next yeast two-hybrid assays (Figure 3).

### 3.5. Yeast Two-Hybrid Screening and Confirmation of the Interactions between pGBKT7-P0 and Screened Prey Plasmids

**Figure 4:**
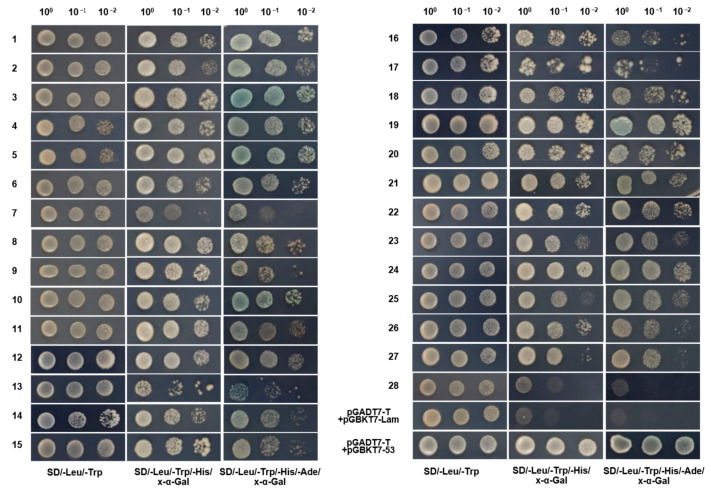
Interaction validation analyses of pGBKT7-P0.

The pGBKT7-P0 bait was co-transformed with the prey plasmids library and cultured on TDO plates and then subsequently transferred onto higher-stringency QDO/X plates where 257 clones could grow and exhibit a blue appearance (Figure S3). To exclude possible false positive clones, 86 screened prey plasmids were extracted from the yeast transformants and then transformed into *E. coli* DH5α for prey plasmids separation and enrichment. Each of the 86 potential positive prey plasmids was again co-transformed into Y2HGold-competent cells with pGBKT7-P0 and cultured on QDO/X plates. After strict screening verification, 28 host proteins were obtained and confirmed to interact with SCYLV P0 (Figure 4).

### 3.6. Sequencing and Analysis of Confirmed True Positive Prey Plasmids

The 28 confirmed true positive prey plasmids were verified by PCR amplification and sent for sequencing with the pGADT7-F/R universal primers to determine sequence information. The sequencing reads were analyzed by using the DNASTAR software and the probable functions were analyzed with the online BLAST tool from the NCBI or Sugarcane Genome Hub (Table S3).

## 4. Discussion

Many studies have indicated that viruses encode different proteins associated with disease symptom formation in plants. For example, the C5 protein of Ageratum leaf curl Sichuan virus (ALCScV) can induce developed severe chlorosis or yellow mosaic in *N. benthamiana* leaves, the C4 protein of ALCScV can induce significant dwarfing and delayed flowering in *N. benthamiana* plants, and the C5 protein of Tomato yellow leaf curl virus (TYLCV) can induce severe mosaic symptoms in *N. benthamiana* leaf [21,22,23]. And as a response to the invading pathogens, plants stimulate HR to defend invading pathogens, and the process is usually associated with the induction of reactive oxygen species (ROS). This type of programed cell death (PCD) is associated with an oxidative burst (including O^2−^ and H_2_O_2_) at the early stage of incompatible interactions between pathogens and plants and is thought to limit or constrain pathogen growth [24]. Cell death increases resistance to biotrophic pathogens but contributes to the proliferation of necrotrophic pathogens [25]. Here, we provide evidence to show that the expression of the SCYLV P0 protein using a PVX-based vector can cause a severe mosaic of leaves and HLR necrosis in *N. benthamiana* plants, and the P0 protein can also promote PVX viral protein accumulation in plants (Figure 2). These results all suggest that P0 may play an important role in the infection of SCYLV as a pathogenic factor. SCYLV usually co-infects with other viruses to aggravate the disease and poses a great threat to sugarcane production. However, the interaction between SCYLV and host proteins is still in the preliminary stage, and the pathogenesis of SCYLV has not been reported. Screening the host proteins interacting with the pathogenic factor P0, revealing its pathogenic mechanism and broadening the cognition of *Polerovirus*, is helpful to conduct research of the molecular mechanism and disease resistance of the virus, and provide a theoretical basis for subsequent disease resistance breeding.

An efficient yeast two-hybrid cDNA library of the sugarcane cultivar ROC22 was constructed in this study. A total of 28 sugarcane host proteins were screened and indicated to interact with the pathogenic factor P0 of SCYLV, which is involved in a variety of biological processes, such as photomorphogenesis, signal regulation, cell development, and the biotic or abiotic stress response (Table S3), among which five were associated with optical paths, NAD(P)H response cells respond to light stimuli, the ultraviolet-B-repressible protein becomes involved in photomorphogenesis, lactoylglutathione lyase responds to light intensity, FTSH 2 is involved in photosynthesis, and photosystem I reaction center subunit XI relates to the absorption and transmission of light energy. Since SCYLV infection causes midrib yellowing and necrosis, and a decline in carbon assimilation because of chlorophyll loss was suspected, the decrease in the contents and ratio of chlorophyll a/chlorophyll b (chla/chlb) was indeed found in the symptomatic leaves [26], and the P0 protein may be the virulence factor leading to the alterations of host photosynthetic apparatus. In addition, SCYLV-infected leaves exhibited ultrastructural changes in Kranz cell chloroplasts and caused earlier senesce [27]. Glucanases are enzymes regulating the size-exclusion limit and permeability of plasmodesmata and play a role in biotic stress [28]. And classes I, II, and III were proved to accumulate following various virus infections and promote viral cell-to-cell spread [28,29]. β-1,3-glucanase A (GluA1) was verified to interact with SCYLV P0 and was dramatically downregulated when infected by SCYLV (Figure S4), which indicated its vital role in viral movement and infection. L-ascorbate oxidase (AO) acts upstream of reactive oxygen species (ROS) and is regulated by a monocot-specific miR528, which negatively regulates viral resistance in rice by cleaving L-ascorbate oxidase (AO) messenger RNA, thereby reducing the AO-mediated accumulation of ROS [30]. Moreover, the movement protein (MP) of Cucumber mosaic virus (CMV) was proved to interact with CsAO4, assist in early viral movement, and reduce the redox defense of the plant during the initial stages of infection [31]. In this study, we found that heterologously expressed SCYLV P0 via a PVX vector could induce H_2_O_2_ accumulation in *N. benthamiana* (Figure 2), the expression level was significantly upregulated in SCYLV-infected sugarcane plants (Figure S4), and P0 was proved to interact with the host L-ascorbate oxidase homolog, which implies that P0 may interfere with host defense by regulating AO expression. It has been shown that Eukaryotic elongation factors (eEFs) are involved in the multiplication of a number of other RNA viruses by directly binding to viral genomic RNA or by interacting with the viral-RNA-dependent RNA polymerase (RdRP) [32,33]. In this study, we found that a viral pathogenic factor P0 of SCYLV can interact with EF1A and may help viral replication. All these interactions between P0 and sugarcane host factors imply its multiple functions besides the RNA silencing suppressor and virulence factor, but also participates in photoperiod, cell development, and other metabolic reactions, which helps to shed light on a comprehensive understanding of the role of P0 and the molecular pathogenesis of SCYLV.

## 5. Conclusions

In summary, we demonstrated that the SCYLV encodes P0 to be a virulence factor and enhances the pathogenicity of PVX in *N. benthamiana*. The SCYLV P0 protein is important for disease symptom formation. In this study, several host proteins that interact with P0 were screened out and identified. To our knowledge, this is the first report of *Polerovirus* P0-interacting proteins. According to the results, we speculate that SCYLV P0 may interfere with plant photosynthesis, endogenous hormone metabolism, plant defense, and other processes to cause abnormal phenotypes and favor viral movement, replication, and other processes, which indicates P0 to be a multifunctional protein during virus infection and helps to shed light on elucidating the pathogenic mechanism of SCYLV. Moreover, the P0 protein may also serve as a potential target in viral resistance breeding.

## Data Availability

The data presented in this study are available in Supplementary Materials.

## Supplementary Materials

The following supporting information can be downloaded at https://www.mdpi.com/article/10.3390/genes14071397/s1, Figure S1: P0 suppresses GFP silencing induced by single-strand RNA and double-strand RNA. (A) Suppression of GFP silencing in 16c transgenic *N. benthamiana* plants. Representative leaf patches were co-infiltrated with *A. tumefaciens* cultures harboring GFP (35S-GFP) and either a pCHF3 vector control (Vec), Sugarcane yellow leaf virus (SCYLV) P0 (P0), or Tomato bushy stunt virus (TBSV) p19 (p19). Infiltrated leaves were photographed at 4 days post inoculation (dpi), under UV light; (B) *N. benthamiana* leaf patches were co-infiltrated with *A. tumefaciens* cultures containing GFP (35S-GFP), dsFP (35S-dsFP), and Vec, P0, or p19, and representative leaf patches were photographed under UV light at 4 dpi. Figure S2: Yeast two-hybrid library of Sugarcane ROC22. (A) Analysis of purified mRNA by 1% agarose gel electrophoresis; (B) the titer of yeast two-hybrid library; (C) detection of inserted fragments of yeast two-hybrid library by PCR followed by agarose gel electrophoresis. Figure S3: Potential P0-interacting positive transformants grown on DQO/X/A plates. Figure S4: Relative expression level detection of representative host factors by qPCR. Relative expression level detection of representative host factors by qPCR. GHD7: transcription factor GHD7; NEP1: NEP1-interacting protein-like 1; GluA1: β-1,3-glucanase A; EF1A: elongation factor 1-α; C2C2-CO: C2C2-CO-like transcription factor. Table S1: Primers used in this study. Table S2: The viruses and corresponding GenBank accession numbers used for phylogenic analysis. Table S3: Analysis results of the confirmed true prey proteins identified using an online BLAST search tool from the NCBI or Sugarcane Genome Hub.
