# Supplementary material for: Screening and Identification of Host Factors Interacting with the Virulence Factor P0 Encoded by Sugarcane Yellow Leaf Virus by Yeast Two-Hybrid Assay
Source: Genes (Basel). 2023 Jul 3;14(7):1397. doi: 10.3390/genes14071397 (PMC10379860; doi:10.3390/genes14071397)
Supplement: Supplementary file 1 [file genes-14-01397-s001.zip › genes-2460989-Supplementary Figures.pdf]

Supplementary Materials:

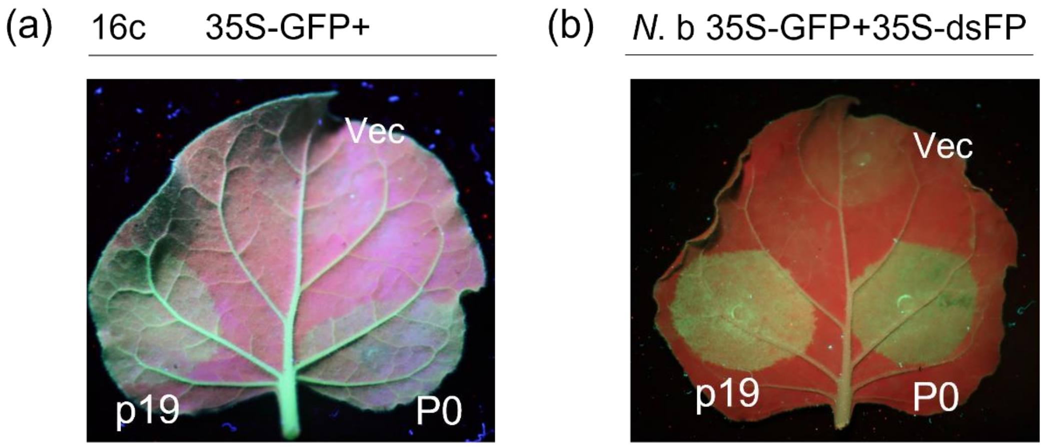

Figure S1. P0 suppresses GFP silencing induced by single-strand RNA and double-strand RNA. (a) Suppression of GFP silencing in 16c transgenic *Nicotiana benthamiana* 16c plants. Representative leaf patches were co-infiltrated with *Agrobacterium tumefaciens* cultures harboring GFP (35S-GFP) and either a pCHF3 vector control (Vec), Sugarcane yellow leaf virus (SCYLV) P0 (P0), or Tomato bushy stunt virus (TBSV) p19 (p19). Infiltrated leaves were photographed at 4 days post inoculation (dpi), under UV light; (b) *N. benthamiana* leaf patches were co-infiltrated with *A. tumefaciens* cultures containing GFP (35S-GFP) and dsFP (35S-dsFP) and Vec, P0 or p19, and representative leaf patches were photographed under UV light at 4 dpi.

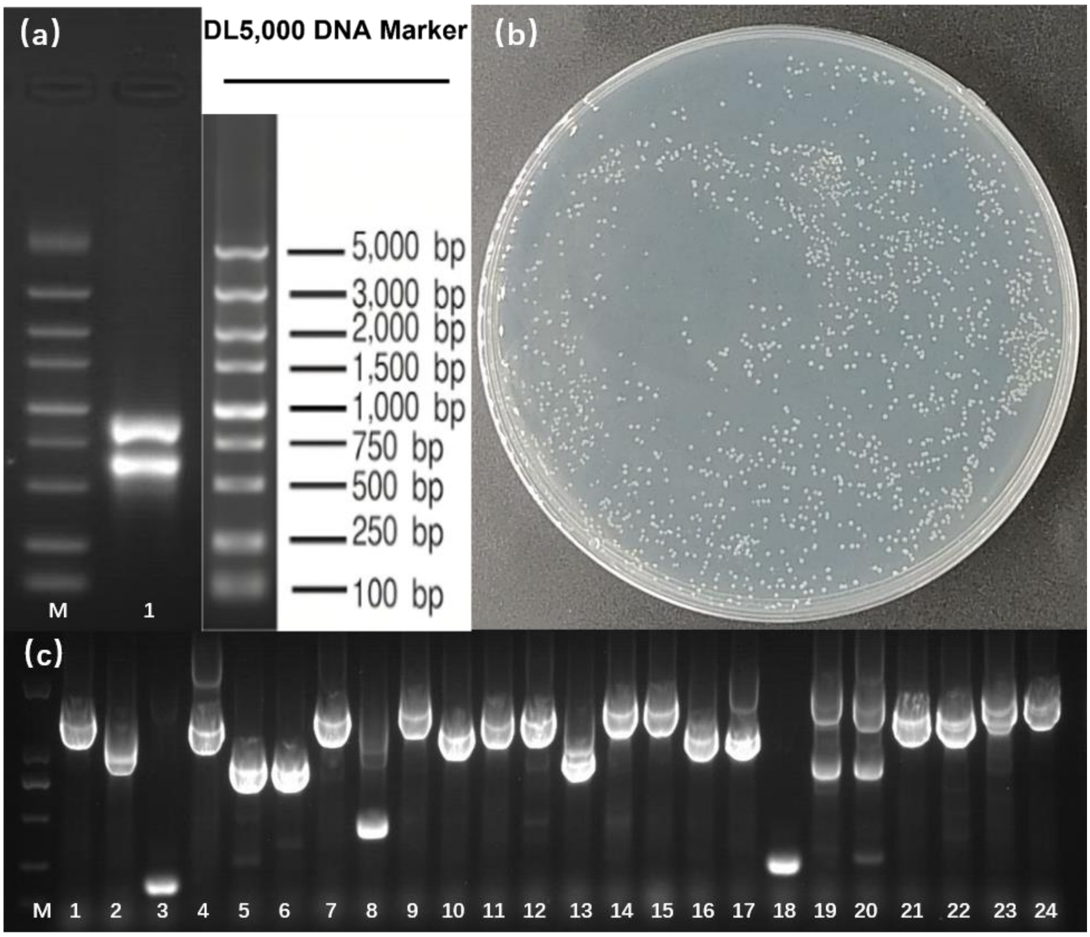

Figure S2. Yeast-two-hybrid library of Sugarcane ROC22. (a) Analysis of purified mRNA by 1% agarose gel electrophoresis, M: DL5,000 DNA Marker; (b) The titer of yeast-two-hybrid library on SD/-Leu agar plates; (c) Detection of inserted fragments of yeast-two-hybrid library by PCR followed by agarose gel electrophoresis.

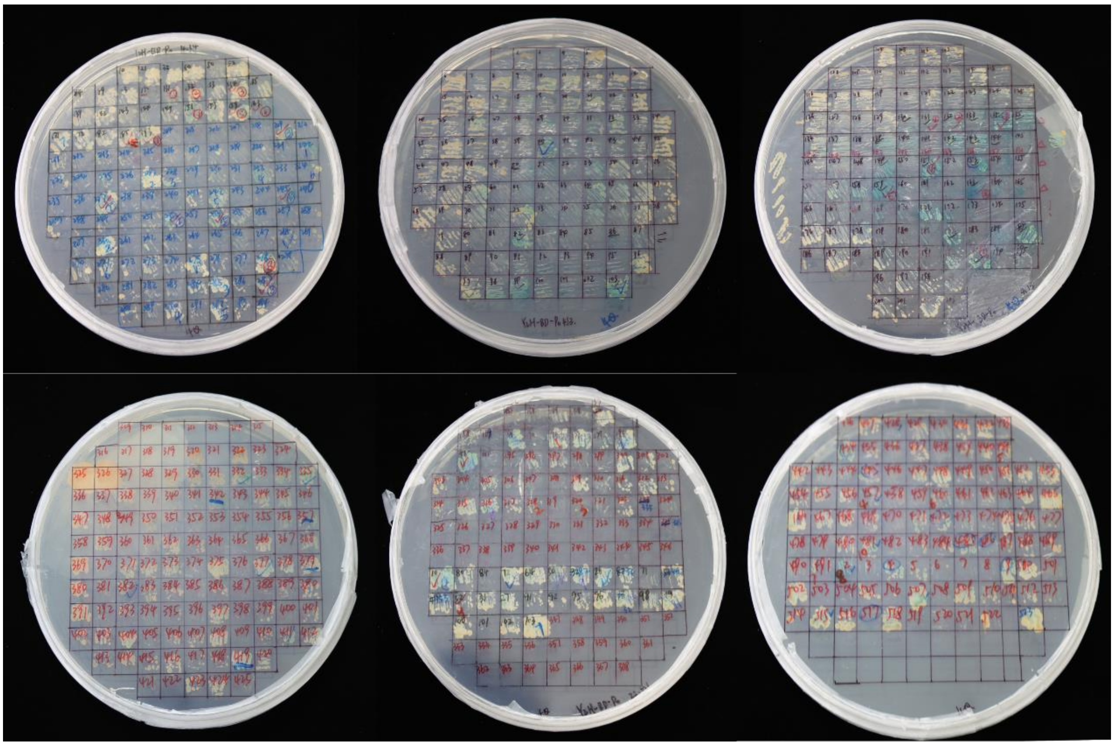

Figure S3. Potential P0-interacting positive transformants grown on DQO/X/A plates.

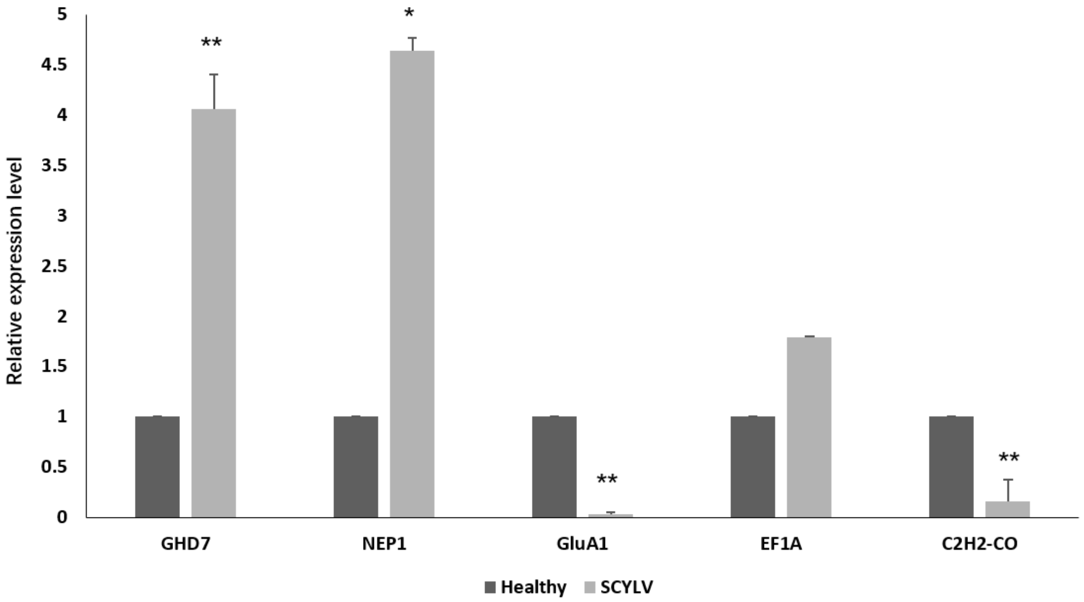

Figure S4. Relative expression level detection of representative sugarcane host factors by RT-qPCR. GHD7: transcription factor GHD7; NEP1: NEP1-interacting protein-like 1; GluA1: beta-1,3-glucanase A; EF1A: elongation factor 1-alpha; C2C2-CO:C2C2-CO-like transcription factor. T-tests were performed to identify statistically significant differences (*P<0.05, **P<0.01). Three individual sugarcane leaf samples were used for RNA extraction and subsequent RT-qPCR detection. All experiments were repeated at least three times.
