# Supplementary material for: Screening and Identification of Host Factors Interacting with the Virulence Factor P0 Encoded by Sugarcane Yellow Leaf Virus by Yeast Two-Hybrid Assay
Source: Genes (Basel). 2023 Jul 3;14(7):1397. doi: 10.3390/genes14071397 (PMC10379860; doi:10.3390/genes14071397)
Supplement: Supplementary file 1 [file genes-14-01397-s001.zip › genes-2460989-Supplementary Tables.pdf]

Table S1. Primers used in this study

Name	Sequence（5'-3'）
SCYLV/F	ACAAAATATATCGGGAGGGAAACC
SCYLV/R	ACTAGGATATACGGGAGGCAGTAC
PVX-F	AGCATCGATTGGCGCGCCT
PVX-R	TTATGGTGGTGGTAGAGTG
SCYLV/P0/BamHI/F	CGGGATCCATATGCTTTTCAACGAATTCTCTG
SCYLV/P0/Sal I/R	CGAGCTCGTCGACCTATACATCATGAGAAGAGGGGC
SCYLV/P0/Cla I/F	CCATCGATATGCTTTTCAACGAATTCTCTG
AD-GAL4/F	TCGATGATGAAGATACCCCA
AD-3AD/R	AGAAATTGAGATGGTGCACG
ScNEP1-q-F	CGATGTGATCTGGAGCTTGCTG
ScNEP1-q-R	CGAAGATGTCGGCGTGGTCT
Scβ1,3-q-F	GGTGATCATGGACGTGCCCAAC
Scβ1,3-q-R	GCCGTGGACACCTTGATGTTCC
ScC2C2CO-q-F	GTCTGGCTCTGCGAGGTCTG
ScC2C2CO-q-R	TCGTTGCTCCCGTCGTCGTC
ScEF1A- q-F	GGCATGGTGGTCACCTTTGG
ScEF1A- q-R	GTGAAGCTGGCAGCCTCCTTG
ScGHD7- q-F	CGTCCAGCGCCACAATCATG
ScGHD7- q-R	GGAGGCGTACCGAATCTGCTTC

The restriction endonuclease site was underline highlighted.

Table S2. The viruses and corresponding GenBank accession numbers used for phylogenic analysis.

Virus	Full name	GenBank accession number	Size (aa)
SCYLV	Sugarcane yellow leaf virus	--	256
CABYV	Cucurbit aphid-borne yellows virus	WBQ21509.1	238
MABYV	Melon aphid-borne yellows virus	YP001949869.1	241
BWYV	Beet western yellows virus	NP 840095.1	238
PeVYV	Pepper vein yellows virus	UIX56023.1	249
TVDV	Tobacco vein distorting virus	ABV01910.1	249
CtRLV	Carrot red leaf virus	YP077185.1	249
PLRV	Potato leafroll virus	NP056746.1	247
CYDV-RPS	Cereal yellow dwarf virus RPS	NP054684.1	246
CYDV-RPV	Cereal yellow dwarf virus RPV	QIE05334.1	256
PABYV	Pepo aphid-borne yellows virus	YP009254737.1	263
SABYV	Suakwa aphid-borne yellows virus	ANH21069.1	269
CLRDV	Cotton leafroll dwarf virus	QLJ58259.1	262
BChV	Beet chlorosis virus	NP114360.1	248
TuYV	Turnip yellows virus	QBG64849.1	249
CpCSV	Chickpea chlorotic stunt virus	YP667837.1	270
MYDV-RMV	Maize yellow dwarf virus RMV	YP008083738.1	261
MYMV	Maize yellow mosaic virus	UNW37587.1	264

Table S3. Analysis results of the confirmed true prey proteins identified using an online BLAST search tool from the NCBI or Sugarcane Genome Hub.

Clone number	Name	Predicted functions	Sequence ID
1	SAM domain family protein	Involved in mediating protein-protein interactions	NM_001156504.2
2	Transcription factor bHLH93	Control height, fertility, chlorophyll level of plants	XM_025956250.1
3	NEP1-interacting protein-like 1	Inducing cell death of dicotyledon	XM_002462714.2
4	beta-1,3-glucanase A (GluA1) gene	Related to plant pathology and stress physiology	KC912762.1
5	elongation factor 1-alpha	Regulates the replication process of various plant RNA viruses	XM_002460814.2
6	L-ascorbate oxidase homolog	negative regulation of defense response to virus, negative regulation of response to salt stress, response to hydrogen peroxide, response to oxidative stress, response to salt stress, response to virus	XM_002459229.2
7	PVR3-like protein	Response to pathogen invasion and environmental stress	NM_001153452.3
8	glyceraldehyde-3-phosphate dehydrogenase 1	Regulating glycolysis and gluconeogenesis metabolic pathway, related to plant growth and stress response	XM_021449348.1
9	Lactoylglutathione lyase	cellular catabolic process, organonitrogen compound catabolic process, response to light intensity	KC857628.1
10	ATP-dependent zinc metalloprotease FTSH 2	Involved in photosynthesis, metabolism of IAA and protein	XM_021449071.1
11	selT-like protein	involved in cell redox homeostasis	XM_002458672.2
12	C2C2-CO-like transcription factor	involvement on the multiple response network regulating the photoperiod and abiotic stress.	KJ727984.1
13	photosystem I reaction center subunit XI	Absorption and transmission of light energy	XM_002457227.2
14	lichenase-2-like	Catalyze decomposing lichenan to D-glucose	XM_021447678.1

15	bidirectional sugar transporter SWEET13	Pollen development，involved in transformation and distribution of sucrose，respond to external stress	XM_002443118.2
16	NAD(P)H-quinone oxidoreductase subunit M	NADH oxidation, cellular response to light stimulus	XM_002446819.2
17	glutamine synthetase (GS1.a)	Nitrogen processes of seed germination and leaf senescence	AY835453.1
18	oxygen-dependent coproporphyrinogen-III oxidase	involvement on chlorophyll biosynthesis in photosynthetic organisms.	XM_002447030.2
19	transcription factor GHD7	Involved in signaling, plant type, stress response, hormone regulation	XM_002464888.2
20	aspartate aminotransferase	aromatic amino acid family biosynthetic process, prephenate pathway, embryo development ending in seed dormancy	XM_021459565.1
21	Ultraviolet-B-repressible protein	Light-sensitive proteins are useful tools to control protein localization, activation and gene expression	Sh_223P16
22	uncharacterized		XM_004979918.2
23	Uncharacterized protein ycf39		XM_002444561.2
24	Putative protein		Sh_239H03
25	similar to Expressed protein		Sh_009I06
26	Conserved hypothetical protein		Sh_215B06
27	Doubtful hypothetical protein		Sh_212O05
28	Doubtful hypothetical protein		Sh_208I03
